# Monogenic Inflammatory Bowel Disease: It's Never Too Late to Make a Diagnosis

**DOI:** 10.3389/fimmu.2020.01775

**Published:** 2020-09-04

**Authors:** Iddo Vardi, Irit Chermesh, Lael Werner, Ortal Barel, Tal Freund, Collin McCourt, Yael Fisher, Marina Pinsker, Elisheva Javasky, Batia Weiss, Gideon Rechavi, David Hagin, Scott B. Snapper, Raz Somech, Liza Konnikova, Dror S. Shouval

**Affiliations:** ^1^Pediatric Gastroenterology Unit, Edmond and Lily Safra Children's Hospital, Sheba Medical Center, Ramat Gan, Israel; ^2^Sackler Faculty of Medicine, Tel Aviv University, Tel Aviv, Israel; ^3^The Genomic Unit, Sheba Cancer Research Center, Sheba Medical Center, Ramat Gan, Israel; ^4^Sheba Medical Center, Wohl Institute of Translational Medicine, Ramat Gan, Israel; ^5^Department of Gastroenterology, Rambam Health Care Campus, Affiliated With Technion-Israel Institute of Technology, Haifa, Israel; ^6^Allergy and Clinical Immunology Unit, Department of Medicine, Tel Aviv Sourasky Medical Center, Tel Aviv, Israel; ^7^Division of Newborn Medicine, Department of Pediatrics, UPMC Children's Hospital of Pittsburgh, Pittsburgh, PA, United States; ^8^Institute of Pathology, Rambam Health Care Campus, Affiliated With Technion-Israel Institute of Technology, Haifa, Israel; ^9^Institute of Nanotechnology and Advanced Materials, Bar Ilan University, Ramat Gan, Israel; ^10^Division of Gastroenterology, Hepatology and Nutrition, Boston Children's Hospital, Boston, MA, United States; ^11^Department of Medicine, Harvard Medical School, Boston, MA, United States; ^12^Pediatric Immunology Service, Edmond and Lily Safra Children's Hospital, Sheba Medical Center, Ramat Gan, Israel; ^13^Pediatric Department Ward A, Edmond and Lily Safra Children's Hospital, Sheba Medical Center, Ramat Gan, Israel; ^14^Jeffrey Modell Foundation Center, Edmond and Lily Safra Children's Hospital, Sheba Medical Center, Ramat Gan, Israel; ^15^Department of Immunology, University of Pittsburgh, Pittsburgh, PA, United States; ^16^Department of Developmental Biology, University of Pittsburgh, Pittsburgh, PA, United States

**Keywords:** CTLA4, common variable immunodeficiency, enteropathy, inflammatory bowel disease, LRBA, monogenic, whole exome sequencing

## Abstract

**Background:** More than 50 different monogenic disorders have been identified as directly causing inflammatory bowel diseases, typically manifesting in the first years of life. We present the clinical course and immunological work-up of an adult patient who presented in adolescent years with an atypical gastrointestinal phenotype and was diagnosed more than two decades later with a monogenic disorder with important therapeutic implications.

**Methods:** Whole exome sequencing was performed in a 37-years-old patient with a history of diarrhea since adolescence. Sanger sequencing was used to validate the suspected variant. Mass cytometry (CyTOF) and flow cytometry were conducted on peripheral blood mononuclear cells for deep immunophenotyping. Next-generation sequencing of the *TCRB* and *IgH* was performed for global immune repertoire analysis of circulating lymphocytes.

**Results:** We identified a novel deleterious c.1455C>A (p.Y485X) mutation in *LRBA*. CyTOF studies demonstrated significant changes in immune landscape in the LRBA-deficient patient, including an increase in myeloid derived suppressor cells and double-negative T cells, decreased B cells, low ratio of naïve:memory T cells, and reduced capacity of T cells to secrete various cytokines following stimulation, including tumor necrosis factor alpha (TNF-α) and interferon gamma (IFN-γ). In addition, this patient exhibited low frequency of regulatory T cells, with a reduction in their CTLA4 expression and interleukin (IL)-10 secretion. Finally, we show marked oligoclonal expansion of specific B- and T-cell clones in the peripheral blood of the LRBA-deficient patient.

**Conclusions:** LRBA deficiency is characterized by marked immunological changes in innate and adaptive immune cells. This case highlights the importance of advanced genetic studies in patients with a unique phenotype, regardless of their age at presentation.

## Introduction

Inflammatory bowel disease (IBD), including Crohn's disease (CD) and ulcerative colitis (UC), are chronic relapsing-remitting inflammatory disorders of the gastrointestinal tract that frequently require the administration of antiinflammatory and immunosuppressive medications (1, 2). Significant progress has been made in the past decade in our understanding of the mechanisms leading to development of IBD. These disorders are thought to develop in genetically susceptible hosts as a result of a dysregulated immune response to microbial dysbiosis, environmental triggers or both (3).

Genetic variants can have different effects on the risk of developing IBD. More than 240 single nucleotide polymorphisms (SNPs) have been identified in genome-wide association studies (GWAS) to confer risk for development of either CD, UC, or both (4). While these relatively common genetic variants explain only a small fraction of IBD heritability, they highlight the importance of key pathways which contribute to intestinal inflammation, such as defects in barrier function, autophagy, and immune responses against microbes (3, 5). In addition, rare deleterious mutations in genes directly causing IBD can be identified (monogenic IBD). In the past decade, and with the development of advanced sequencing techniques, more than 50 different monogenic disorders causing IBD have been described (6, 7). In most cases of monogenic IBD, the phenotype is severe and the gastrointestinal inflammation is often accompanied by susceptibility to recurrent infections. Identification of a monogenic cause can have a marked impact on a patient's care, since “non-conventional” targeted therapies can be applied. For example, we have shown that anakinra, an interleukin (IL)-1 receptor antagonist, can ameliorate colitis in patients with IL-10 receptor (IL-10R) deficiency (8) and similarly, hematopoietic stem cell transplantation (HSCT) is the treatment of choice for several of these monogenic disorders [e.g., IL-10R deficiency (9, 10), chronic granulomatous disease (11), Wiskott–Aldrich syndrome (12)].

In the majority of cases, monogenic IBD presents in the first years of life. Very early onset IBD (VEO-IBD) is defined as development of intestinal inflammation prior to the age of 6 years, and infantile onset IBD is defined as onset at age <2 years (6). Monogenic disorders are identified in up to 12% of VEO-IBD cases and in up to 41% of infantile-onset IBD patients (13–17). Fortunately, it has become quite standard in many centers to refer young patients with VEO-IBD for advanced genetic studies by either sequencing of defined gene panels, whole exome sequencing (WES), or even whole genome sequencing. However, physicians are less aware of the possibility that these rare disorders can also present in adults. Here we present the clinical course and immunological workup of an adult patient who presented in adolescent years with an atypical clinical phenotype and was diagnosed with a monogenic disorder more than two decades later, with important therapeutic implications.

## Methods

### Whole Exome Sequencing

The study was approved by the local IRB committee at Sheba Medical Center. Informed written consent was obtained from the patient. Whole exome sequencing was performed using an Agilent v5 Sureselect capture kit and Illumina 2500 sequencing technology. Paired end reads (2 × 100 bp) were obtained, processed, and mapped to the genome. The average sequencing depth of the target region is 92 × , with 95.63% of bases reached at least 10 × coverage. We used the BWA mem algorithm (version 0.7.12) (18) for alignment of the sequence reads to the human reference genome (hg19). The HaplotypeCaller algorithm of GATK version 3.4 was applied for variant calling, as recommended in the best practice pipeline (19). KGG-seq v.08 was used for annotation of identified variants (20) and in-house scripts were applied for filtering based on family pedigree and local dataset of variants detected in previous sequencing projects. Likely pathogenicity was assessed if the variant was truncating (splicing or nonsense) or missense; in-frame indels were considered if they were predicted to be pathogenic by online prediction tools including PolyPhen-2, SIFT, CADD, and MutationAssessor.

### Sanger Sequencing

Exon 11 of lipopolysaccharide (LPS)-responsive and beige-like anchor (*LRBA*) gene were amplified and sequenced by the Sanger method. Briefly, polymerase chain reaction (PCR) amplification was performed using three sets of the following primers:

Fw-5′-GCTTCTGTTAAACACATGGAATGTC-3′

Rw-5′-ACAACAAAACCTGAAAGGCAAA-3′

The PCR reaction took place in a 25-μL volume containing 50 ng of DNA, 10 ng of each primer, 1.5 mM dNTPs, in 1.5 mM MgCl_2_, PCR buffer, with 1.2 units of Taq polymerase (Bio-Line, London, UK). After an initial denaturation of 5 min at 95°C, 30 cycles were performed (94°C for 30 s, 60°C for 30 s, and 72°C for 30 s), followed by a final extension of 10 min at 72°C. PCR amplicons were sequenced in both directions using a commercial sequencing service [Hy Laboratories, Ltd. (Hylabs) Park Tamar Rehovot, Israel].

### Mass Cytometry Studies

Peripheral blood mononuclear cells (PBMCs) from the LRBA-deficient patient and two healthy controls, obtained from the Vitalent blood bank at the University of Pittsburgh as leftover buffy coat, either underwent a 4-h incubation at 37°C in 5% CO_2_ with GolgiStop/GolgiPlug (BD Biosciences, San Jose, CA, USA) prior to staining (baseline conditions) or stimulated for 4 h with 50 ng/mL of phorbol myristate acetate (PMA) and 500 ng/mL of ionomycin with GolgiStop/GolgiPlug. Next, cells were stained with a panel of metal-tagged antibodies targeting markers of major immune cell lineages and various cytokines (Supplemental Table 1) per a previously published protocol (21). The samples were run on Helios2 mass cytometer (Fluidigm, San Francisco, CA, USA). FCS files obtained were analyzed with premium Cytobank (Santa Clara, CA, USA) software and pregated on CD45^+^/viable/single/DNA^+^ events before initiating analysis. Normalization beads were used and gaited out of the analysis. The data were imported into Cytofkit (22) and automatically clustered with Phenograph (23) and clusters were manually annotated based on a marker expressed in the individual clusters. Markers used for clustering highlighted in blue (Supplemental Table 1). The data were visualized with *t*-SNE plots. The cluster percentage (% of CD45^+^ viable single events) was computed and plotted for comparison between patient groups using Prism8 software. Cytokine levels were analyzed either by median intensity of the marker within particular clusters or by percent-positive events within that cluster.

### Flow Cytometry Staining

PBMCs were thawed and left overnight to recover in complete medium at 37 °C. The next day, cells were surface stained for 30 min at room temperature using the following antibodies: CD25 PE/Cy7 (clone BC96), CD4 Pacific Blue (clone RPA-T4M), and CD127 PE (clone A019D5). Cells were then washed, fixed, and permeabilized with True-Nuclear™ Transcription Factor Buffer Set (BioLegend, San Diego, CA, USA) according to the manufacturer's recommendations and stained intracellularly and intranuclearly using the following antibody mix: FOXP3 AF®488 (clone 259D), Helios AF®647 (clone 22F6), and CTLA4 PerCP/Cy5.5 (clone BNI3). All antibodies were purchased from BioLegend. Cells were acquired using BD FACSCanto II flow cytometer and analyzed using FlowJo software (V10.0, TreeStar).

### Immune Repertoire Studies

Next-generation sequencing (NGS) of the *TCRB* and *IgH* were performed to define the global T-cell receptor (TCR) and B-cell receptor (BCR) immune repertoire. Briefly, 2 of μg genomic DNA was extracted from blood (Wizard kit, Promega, WI, USA) and used for *TRB* and *IgH* repertoire library generation. Amplified sequences were subjected to high-throughput sequencing using Illumina technology. Graphical presentation of the repertoire was presented using hierarchical tree maps using the Treemap software (www.treemap.com). Shannon's *H*, which measures the overall diversity in a given population (24), and takes into account the number of unique sequences (richness of the repertoire) and how evenly the sequences are distributed, was calculated using the following formulas:

Shannon's *H* = $-\overset{R}{\underset{i=1}{\sum }}\;p_{i}\;In\;p_{i}\;$

where

*R* = total templates

*i* = unique rearrangements

*p*_*i*_ = proportion of the total sequences belonging to the *i*th unique rearrangement.

## Results

### Case Description

A 37-years-old male patient was referred to our clinic from another hospital for evaluation of chronic intermittent diarrhea. The patient was of Druze descent, born to consanguineous parents (parents were double first-degree cousins). Family history was significant for two brothers who died of gastric cancer at the ages of 17 and 27 years, a sister who died of leukemia at 13 years of age, and an older brother with celiac disease.

**Figure 1:**
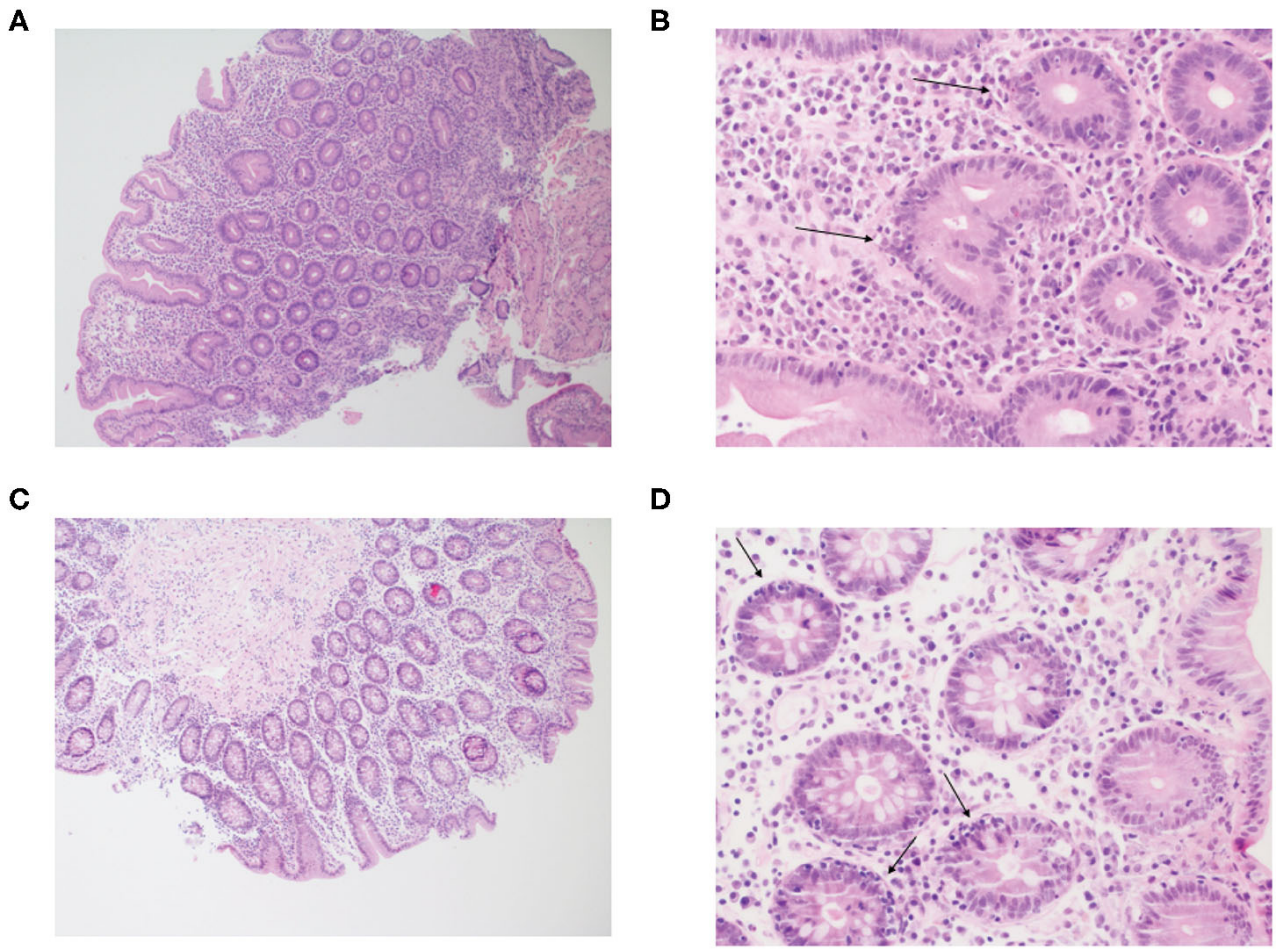
Inflammatory cell infiltration of the small and large intestine in the patient with LRBA deficiency. Figure displays hematoxylin and eosin (H&E) stain at **(A)** ×100 and **(B)** ×400 of duodenal biopsies. The mucosa shows flattened villi and hyperplastic crypts, with an increase in plasma cells in the lamina propria. Crypt epithelium shows increased intraepithelial lymphocytes (arrows). Next, an H&E stain of the transverse colon is presented at **(C)** ×100 and **(D)** ×400. The structure of the colonic mucosa is preserved. The stroma is infiltrated by mononuclear inflammatory cells and the crypt epithelium shows increased intraepithelial lymphocytes (arrows), similar to the duodenal biopsy. The lamina propria shows increase in plasma cells.

The patient initially presented at the age of 15 years with recurrent diarrhea and was diagnosed with celiac disease based on a positive anti-tissue transglutaminase and histologic findings on duodenal biopsies. He started a gluten-free diet with good compliance and had no symptoms for ~13 years. At 28 years of age severe watery diarrhea recurred, leading to electrolyte imbalance, vitamin D and vitamin B_12_ deficiency, secondary hyperparathyroidism, and osteoporosis. HLA-DQ2/DQ8 typing was negative, ruling out celiac disease. Multiple gastroscopies were conducted over the following years revealing atrophic gastritis, duodenal mucosa with cobblestone pattern, and mildly nodular and flattened duodenal folds. Colonoscopy was macroscopically normal. Gastric biopsies showed chronic atrophic gastritis, without identification of Helicobacter pylori. Duodenal biopsies demonstrated villous atrophy and crypt hyperplasia, similar to that seen in celiac disease; however, increased intraepithelial lymphocytes were seen only in the crypt epithelium and not in the surface epithelium (Figures 1A,B). In addition, an increase in lamina propria plasma cells was noticed. *Giardia* was not identified. Colonic biopsies showed changes similar to those of the duodenal biopsies with increased intraepithelial lymphocytes in the crypt epithelium, but not in the surface epithelium, and an increase in plasma cells in the lamina propria (Figures 1C,D).

Aside from his gastrointestinal symptoms, the patient developed in his late 20s symmetric polyarthritis of small and large joints and sensory-motor neuropathy of unclear etiology, manifesting as distal limb weakness and muscle atrophy. He also exhibited recurrent episodes of hepatitis with no etiology found despite extensive workup that included autoimmune hepatitis serologies and a liver biopsy. Importantly, the patient did not suffer from major infections, aside from recurrent sinusitis and tonsillitis. There were no signs of chronic lung disease. Over the years, the patient was treated with multiple courses of steroids (prednisone and budesonide) with good clinical response of both diarrhea and arthritis. Methotrexate was effective only for a short period of time and was therefore discontinued.

On physical exam at our institution, the patient appeared thin (BMI 19.2). His lungs were clear to auscultation. However, clubbing was noted. His neurological examination was notable for tremor and bilateral drop foot, along with atrophy of the plantar region of both hands. Hypopigmented lesions were noted on the skin, suggesting a diagnosis of vitiligo. Both chest CT and brain MRI were normal, without bronchiectasis or white matter lesions, respectively.

Immunological workup demonstrated normal IgG (1,507 mg/dL) and IgM (46 mg/dL) levels, and slightly decreased IgA (54.0 mg/dL, normal range 70–400) level. Lymphocyte subset analysis was normal, except for decreased frequency of CD20^+^ B cells (2%, normal range 5–25%; absolute number 46 cells/μL, normal range 50–300 cells/μL). To define B-cell memory, specific antibody responses against childhood vaccines were obtained. The patient exhibited good titers against poliovirus, hepatitis A, measles, rubella, and diphtheria and borderline levels against tetanus. He was tested negative for hepatitis B and mumps. Importantly, the patient was not on intravenous gamma-globulin replacement therapy at the time the samples were obtained. Finally, we measured isohemagglutinin levels, which indicate the ability of an individual to develop antibodies to polysaccharide antigens. Since the patient's blood type is O^+^ we could measure both anti-A and anti-B antibody levels. Titers of both were positive (1:32), suggesting that the patient has an ability to develop antibodies to polysaccharide antigens.

### Identification of a Novel *LRBA* Mutation

**Figure 2:**
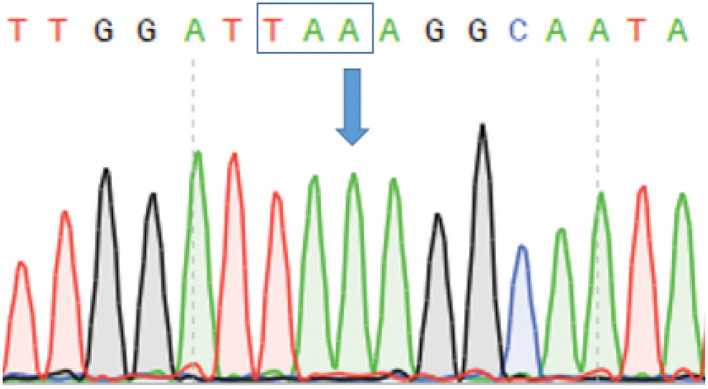
Sanger sequencing confirming stop-gain mutation in *LRBA*. Figure depicts chromatogram of *LRBA* Sanger gene sequencing. Arrow indicates the position of the mutation (c.1455 C>A) resulting in a stop codon indicated by a box.

Family history and parental consanguinity suggested an autosomal recessive genetic disorder. Single WES was performed focusing on homozygous or compound heterozygous variants. Genetic analysis yielded 8274 homozygous variants that affect protein sequences. This list of variants was subsequently reduced to 21 rare homozygous and hemizygous variants (Supplemental Tables 2, 3), by filtering out variants present in ≥0.01 of our in-house exomes (*n* ~ 1,870) and variants present with a minor allele frequency (MAF) ≥ 0.01 in either the 1000 Genomes Project (1 KG; https://www.internationalgenome.org/1000-genomes-browsers) or dbSNP 135 database, the NHLBI Exome Sequencing Project (ESP) (http://evs.gs.washington.edu/EVS/) or gnomAD database (https://gnomad.broadinstitute.org/). A c.1455C>A (p.Y485X) stop-gain mutation (NM_006726) in the *LRBA* gene was identified and was validated by Sanger sequencing (Figure 2). The stop-gain mutation was found in exon 11 (out of 57) and was not reported previously. The variant was not found in gnomAD Database nor in dbSNP, 1000G and ESP6500, nor in our in-house database.

### Aberrant Immune Landscape in LRBA Deficiency

**Figure 3:**
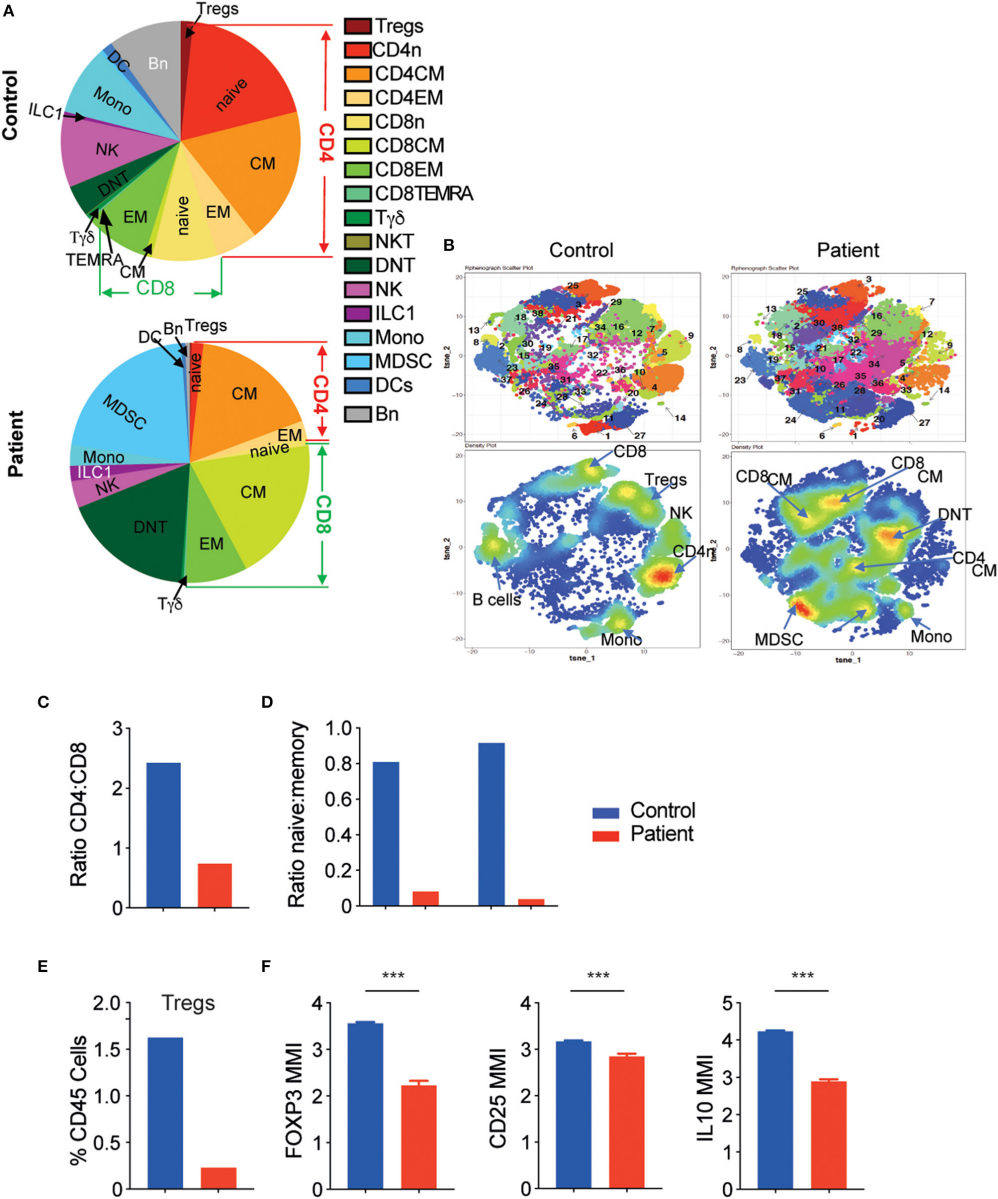
The LRBA-deficient patient exhibits significantly altered immune landscape. **(A)** Main immune subtypes as a proportion of total leukocytes in control and patient samples. **(B)** tSNE map generated after Phenograph clustering of all samples combined. Top row: cluster map. Bottom row: density plots. **(C)** Ratio of CD4^+^ to CD8^+^ T cells and **(D)** ratio of naïve:memory T cells in control and patient samples. **(E)** Tregs as percent of total leukocytes. **(F)** Mean metal intensity (MMI) of FOXP3, CD25, and IL-10 in patient and control Tregs. ***P < 0.001. CM, central memory; DC, dendritic cells; DNT, double negative T cells; EM, effector memory; ILC, innate lymphoid cells; MDCS, myeloid derived suppressor cells; Mono, monocytes; n, naïve; NKT, natural killer T cells; Tregs, regulatory T cells.

To obtain a comprehensive overview of the effect of aberrant LRBA expression on different immune populations in the blood, we performed CyTOF analysis of PBMCs obtained from the patient (two blood samples at different timepoints), and compared the profiles to two healthy control subjects obtained from the blood bank. Cells from all samples were clustered together by similar phenotypes using Phenograph automated clustering that generated 38 unique clusters (Figure 3A and Supplemental Figure 1); cluster identity was defined through the marker expression of associated heatmap (Supplemental Figure 1). Clusters of similar phenotypes were then grouped together to obtain a picture of the immune landscape differences.

As can be seen in Figures 3A,B and Table 1, there were marked shifts in both innate and adaptive immune cells in the patient compared with controls. Looking at innate immune cells, there was a reduction in monocytes (clusters 1 and 27, CD19^−^CD14^+^, Figures 3A,B, Supplemental Figure 1) with a striking increase in myeloid-derived suppressor cells (MDSCs, clusters 24 and 28, CD14^−^HLA-DR^−^CD11c^−^CD11b^+^) in the patient compared with the control (20.9. vs. 0.4%, Figures 3A,B, Supplemental Figure 1, Table 1). B cells were also decreased in the patient (clusters 8 and 23, CD19^+^, 9.6% control vs. 0.6% patient, Figures 3A,B, Supplemental Figure 1, Table 1), consistent with the lymphocyte subset analysis described earlier.

**Table 1:** Percent of various leukocyte populations and accompanying normal values.

	**Control**		**Patient**		**Reference values**		**Parent population**
	**% Leukocytes**	**% Parent population**	**% Leukocytes**	**% Parent population**	**% Leukocytes^a^**	**% Parent population^b^**	
Tregs	1.6	3.7	0.2	1.1		3 (1–6)	CD4
CD4 naïve	18.9	44.7	1.6	7.6		43 (18–66)	CD4
CD4 CM	17.8	40.6	17.0	81		33 (19–52)	CD4
CD4 EM	5.5	12.6	2.2	10.4		17 (7–30)	CD4
Total CD4	43.9		21.0		25–60		
CD8 naïve	8.6	31.1	1.1	1.9		36 (8–67)	CD8
CD8 CM	1.0	3.5	19.1	34.3		10 (3–22)	CD8
CD8 EM	8.5	30.5	8.2	14.7		19 (6–39)	CD8
CD8 TEMRA	0.3	1	0.0	0		9 (2–21)	CD8
Total CD8	18.4		28.3		5–30		
Total T cells	62.2		49.4		55–84		
Tγδ	0.6		0.3				
NKT	0.1	1.4	0.3	0.42		6 (1–15)	CD3
DNT	4.1	5.7	17.3	22.5		7 (2–21)	
NK	9.4		3.6		10–30		CD3
ILC1	0.5		2.0				
Mono	9.0		2.7				
MDSC	0.4		20.9				
Total Mono	9.5		23.6		5–10		
DCs	1.4		0.6		1–2		
B cells	9.6		0.6		5–10		

Since there are no CyTOF “normal” values, we show reference values obtained from

a www.miltenyibiotec.com and Bisset et al. (25) and from

b *Apoil et al. (26). Red color indicates values that are higher than reference range and blue color indicates values that are lower than the reference range. CM, central memory; DC, dendritic cells; DNT, double negative T cells; EM, effector memory; ILC, innate lymphoid cells; MDCS, myeloid derived suppressor cells; Mono, monocytes; NKT, natural killer T cells; Tregs, regulatory T cells*.

Moreover, our analyses demonstrate that the patient had increased abundance of memory CD8^+^ T cells (clusters 25, 38, 30, 21, and 37), accompanied by a marked decrease in naïve CD4^+^ T cells (cluster 4, Figures 3A,B). Consequently, the ratio of CD4:CD8 was substantially lower in the patient vs. the control (Figure 3C). In addition, the ratio between naïve and memory T cells was also markedly lower for both CD4^+^ and CD8^+^ T cells among the patient (Figure 3D). Finally, double negative T cells (DNT, cluster 19, CD3^+^CD4^−^CD8^−^) were increased in the LRBA-deficient patient compared with the control (17.3 vs. 4.1%, Figures 3A,B).

**Figure 4:**
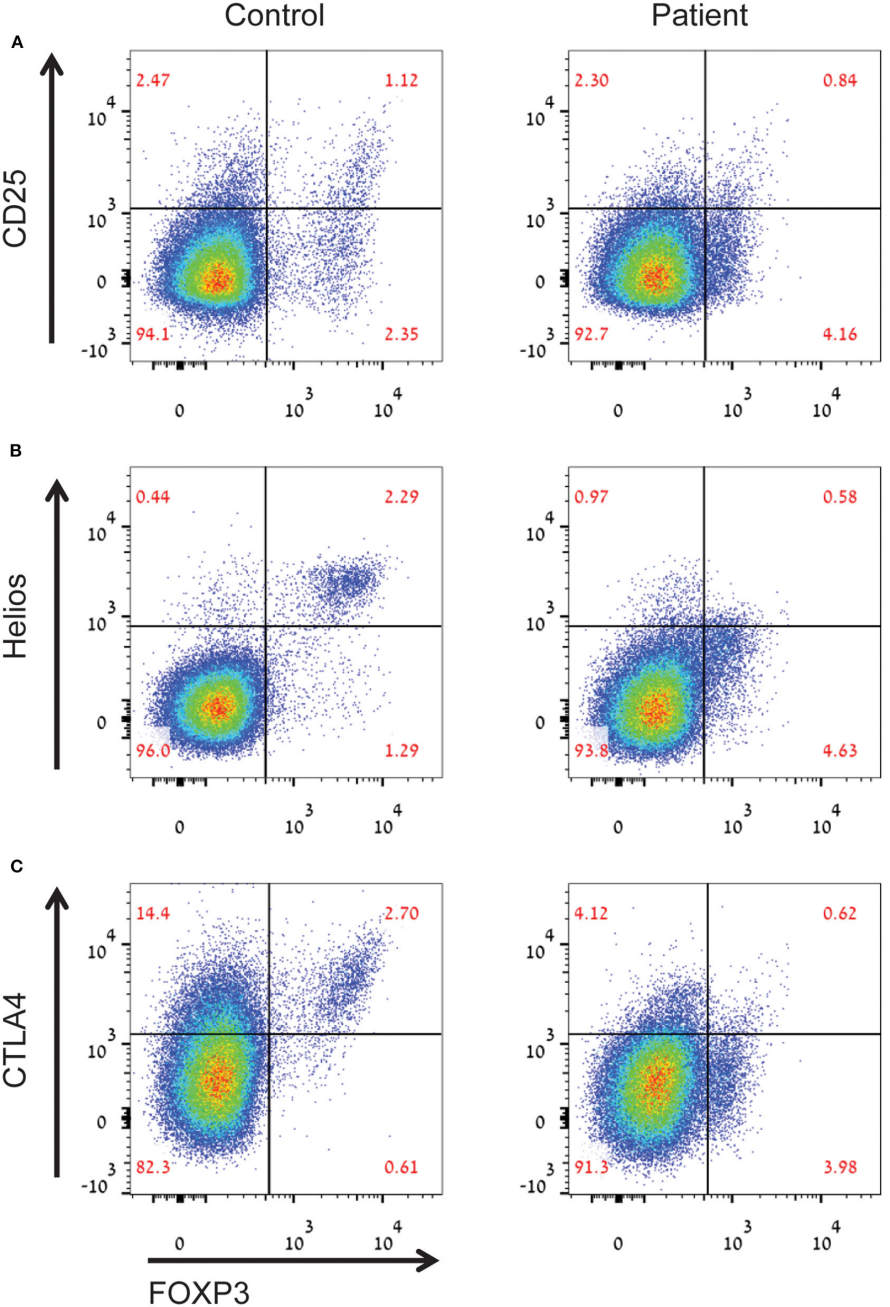
Aberrant Treg phenotype in the LRBA-deficient patient. Figure depicts **(A)** decreased percentages of CD25^high^FOXP3^+^ Tregs, **(B)** decreased levels of helios^+^ inducible Tregs, and **(C)** low levels of CTLA4 in Tregs in the LRBA-deficient patient, compared with control. Cells gated on CD4^+^ T cells.

An important observation in CyTOF analysis was very low percentages of FOXP3^+^ T regulatory cells (Tregs, cluster 7) in the LRBA-deficient patient (Figure 3E). Among Tregs, expression levels of FOXP3, CD25 and also IL-10, an important immunoregulatory cytokine, were reduced in the patient's sample (Figure 3F). We next validated our CyTOF Treg findings by flow cytometry. Analysis revealed low frequency of FOXP3^+^ Tregs in the LRBA-deficient patient, and specifically reduced numbers of Helios-positive Tregs (Figures 4A,B). Importantly, we observed low expression of CTLA4 in Tregs from the index patient (Figure 4C), corroborating previous findings (27). Collectively, our studies identified marked changes in the immune landscape in this LRBA-deficient patient and suggest an immunological IPEX-like phenotype.

**Figure 5:**
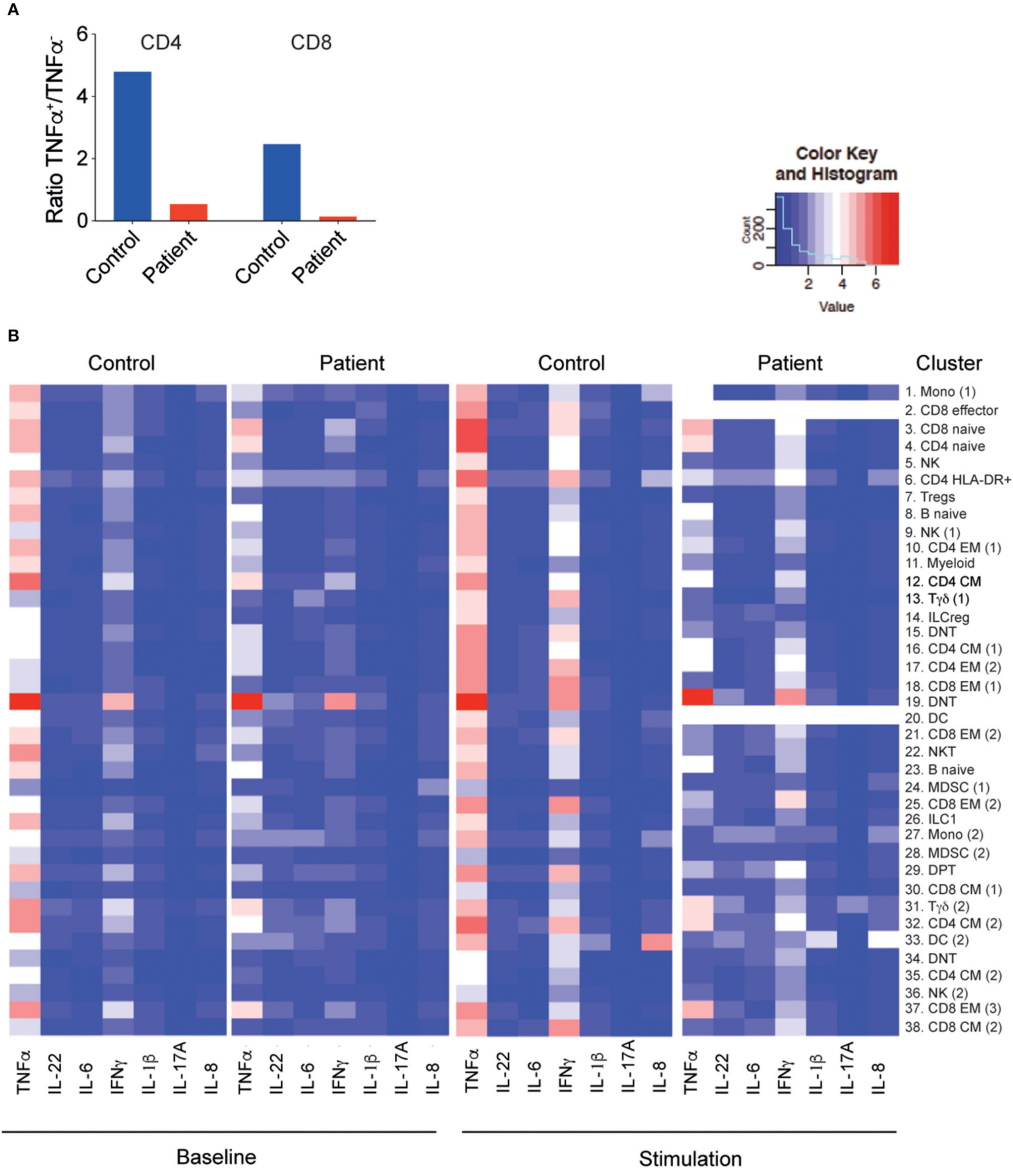
Decreased cytokine production by the LRBA-deficient patient. **(A)** Ratio of memory T cells producing TNF-α to those not producing any TNF-α, without stimulation. **(B)** Heatmap of cytokine expression under baseline conditions and with PMA/I stimulation in clusters associated with Figure 3B. PMA/I, phorbol 12-myristate 13-acetate and ionomycin.

Next, we examined by CyTOF the cytokine production of PBMCs at baseline or in response to stimulation for 4 h with PMA and ionomycin. The overall baseline cytokine production, especially in T cells, was significantly lower in the LRBA-deficient patient than in controls, with a reduced ratio of tumor necrosis factor (TNF)-α^+^ and TNF-α^−^ T cells (Figure 5A, Supplemental Figure 1). Moreover, conventional T-cell clusters (clusters 2, 3, 6, 10, 12, 16, 17, 18, 21, 25, 32, and 38) of the LRBA-deficient patient produced less TNF-α and interferon (IFN)-γ in response to stimulation than the control subject (Figure 5B, Supplemental Figure 1). Interestingly, cytokine production of the DNT (cluster 19) was preserved in the LRBA-deficient patient and the γδ T cells (cluster 31) had higher IL-17A expression in the LRBA-deficient patient than in the control (Figure 5B, Supplemental Figure 1).

### Restricted T- and B-Cell Receptor Repertoire in LRBA Deficiency

**Figure 6:**
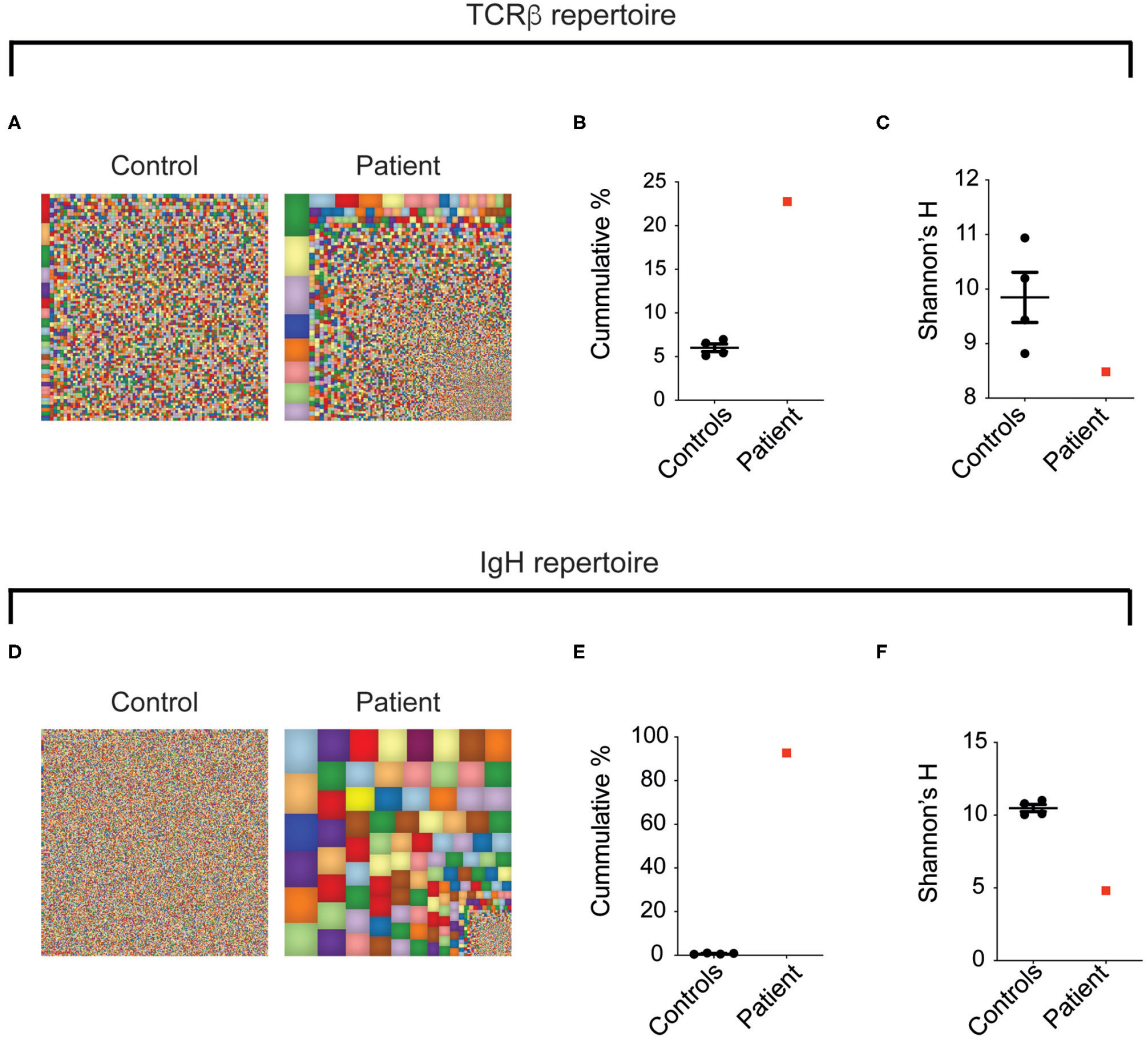
Oligoclonal expansion of T- and B-cell clones in the LRBA-deficient patient. Figure displays **(A,D)** representative Treemap images, **(B,E)** cumulative percentages of the top 100 clones, and **(C,F)** Shannon's *H* analysis of *TCRB* and *IgH*, respectively, assessed by NGS of peripheral blood.

To further define the global landscape of T and B cells in this patient, circulating TCR-β and BCR repertoires were examined using NGS and compared to four healthy controls. For a broad overview of all T cells, we used Treemaps whereby each square represents a different clone and the size correlates with its frequency. As can be seen in Figure 6A, the TCR repertoire in the patient was oligoclonal, with expansion of specific clones. To follow, the cumulative sum of the 100 most abundant clones was found to be strikingly higher in the patient, compared with the controls (22.8% vs. 6.0 ± 0.4, Figure 6B). In addition, Shannon's *H* index, which measures the overall diversity in a given population, revealed a prominent decrease in the patient's diversity (8.4 vs. 9.8 ± 0.5, Figure 6C). Analysis of the BCR repertoire showed marked oligoclonal expansion, as can be seen in Treemap images (Figure 6D), cumulative percentages of the top 100 clones (Figure 6E) and Shannon's *H* index of diversity (Figure 6F). Overall, our data highlight that the aberrant landscape of adaptive immune cells is accompanied by marked expansion of T cells and to a greater extent B cells, which might have a role in the pathogenesis of this multisystemic disorder.

## Discussion

Most monogenic disorders causing IBD-like phenotypes typically manifest at a very young age (6), and therefore sequencing efforts have been focused mainly in patients with IBD younger than 6 years, and especially those who present in the first 2 years of life. Here we describe a patient who presented with recurrent diarrhea resulting from chronic enteropathy at adolescence, along with additional autoimmune features that evolved over the years and was finally diagnosed at adulthood with LRBA deficiency. Interestingly, the patient did not display a previous history of recurrent infections, besides mild recurrent sinusitis and tonsillitis.

LRBA, a member of the BEACH-WD40 protein family, has a role in regulating cell surface expression of CTLA4, a key inhibitor of T-cell proliferation (27). The loss of LRBA protein expression, by homozygous or compound heterozygous mutations, was found to lead to reduced levels of CTLA4 protein in FOXP3^+^ Tregs (as we show for our patient) and in activated conventional T cells (27, 28), thus causing a primary immunodeficiency with features overlapping those of CTLA4 deficiency (29).

Interestingly, from an immunological point of view, LRBA-deficient patients can present with different phenotypes (30). These include an IPEX-like phenotype, with decreased frequency of FOXP3^+^ Tregs (27, 31) (as in the patient we describe); autoimmune lymphoproliferative syndrome (ALPS)-like (32), characterized by elevated double negative T cells, which were also seen in our patient; and raised serum Fas ligand levels. Another common presentation is of common variable immunodeficiency (CVID) (33), characterized by hypogammaglobulinemia and lower B cell numbers, again observed in our patient. Another interesting finding revealed from our CyTOF analysis was a marked increase in MDSCs, which has also been demonstrated in patients with CVID and thought to contribute to immune dysregulation (34). MDSCs are known to suppress T-cell activation and are increased among PBMCs of neonates (35) and in pathologic conditions, such as chronic inflammation and cancer (36). Consistent with this, we observed a suppression of baseline cytokine production and decreased activation in response to stimulation in the patient reported. Furthermore, we observed substantial dysregulation in the CD4:CD8 T cell ratio with increased memory T cells in the patient, consistent with previously described phenotypes observed in LRBA deficiency (33).

LRBA deficiency is a highly heterogenic disease that can manifest as immunodeficiency with recurrent infections (mainly sinopulmonary), IBD, autoimmunity, arthritis, or lymphoproliferation with lymphadenopathy and organomegaly (33, 37–40). Importantly, LRBA deficiency was linked to several malignancies, including lymphoma, gastric adenocarcinoma, as well as one report of melanoma (32, 33, 41). In addition, the age of onset also varies between different studies, starting from infancy to adulthood. Therefore, these factors can make the diagnosis challenging and a high index of suspicion is required. The patient we present had several features consistent with previous reports, including autoimmunity, enteropathy, hepatosplenomegaly, sinusitis, arthritis and clubbing (Table 2) (37). In addition, he presented in adolescent years, as did some of the other LRBA-deficient patients reported before (33). Interestingly, several studies have failed to demonstrate a genotype–phenotype correlation, and patients with the same mutation can have different clinical manifestations (33, 42, 43).

**Table 2:** Comparison of the clinical phenotype of the index patients to other LRBA-deficient patients.

**Manifestation**	**Index patient**	**LRBA-deficient patients (%)**
Autoimmunity	+	82
Enteropathy	+	63
Splenomegaly	Mild (13.6 cm on ultrasonography)	57
Recurrent pneumonias	–	49
Chronic diarrhea	+	48
Lymphadenopathy	–	43
Hepatomegaly	+ (17 cm on ultrasonography)	40
Failure to thrive	–	37
Interstitial lung disease	–	28
Sinusitis	+	24
Bronchiectasis	–	23
Otitis media	–	21
Clubbing	+	19
Arthritis	+	18
Granulomatous lesions	–	14
Allergy and asthma	–	14
Candidiasis	–	10
Septicemia	+ (following perforated appendicitis)	9
Abscess	–	6
Malignancy	–	6
Meningitis	–	5
Osteomyelitis	–	1

*The clinical manifestations of the reported patient were compared to 103 patients with LRBA deficiency reported elsewhere (37)*.

Although the patient we report developed over the years a multisystemic disease, the main phenotype was chronic intermittent diarrhea, initially thought to be linked to celiac disease. Diarrhea in patients with LRBA deficiency is common (40, 43). Some of these subjects can present with enteropathy (43), as commonly seen in patients with CVID. However, other patients manifest with a CD-like or UC-like intestinal inflammation (13, 14, 44), while perianal abscesses have also been reported (45).

The treatment of LRBA deficiency is complex, since on the one hand these patients have an immunodeficiency with increased risk of infections, but on the other hand can develop chronic intestinal inflammation and autoimmunity requiring immunosuppressive therapies. Many of these patients were treated over the years, prior to the genetic diagnosis, with different immunosuppressive regimens, including corticosteroids, cyclosporine, thiopurines, mycophenolate mofetil, tacrolimus, sirolimus, infliximab, rituximab, and hydroxychloroquine (33, 40). Nevertheless, advances in understanding the pathogenesis of this disorder have facilitated administration of targeted therapies. Abatacept, a CTLA4 fusion protein, was shown to be effective in ameliorating different autoimmune features among patients with LRBA deficiency (27, 46), including intestinal inflammation, and can be administered alone or in combination with sirolimus. In addition, these patients are frequently treated with immunoglobulin replacement, with or without prophylactic antibiotics. Our patient had normal levels of immunoglobulins and importantly good specific antibody responses; therefore, he was not put on immunoglobulin replacement therapy.

Hematopoietic stem cell transplantation (HSCT) is suggested as a curative treatment for LRBA-deficient patients suffering from severe and refractory clinical manifestations, with good outcomes in different studies (47, 48). Interestingly, in some patients, residual autoimmune features persisted after HSCT (47), which may suggest that LRBA has a role in non-immune cells as well.

Following diagnosis, our patient was recommended to start abatacept treatment. The question of HSCT is more complex, given his age and limited data regarding success rates of HSCT in adult patients with primary immunodeficiencies. Nevertheless, in this specific patient, with such a significant family history of malignancies, atrophic gastritis, and chronic enteropathy, we do believe that HSCT should be strongly considered, while appreciating the risks. An alternative option is to continue abatacept for an extended period of time, if effective, along with close monitoring including yearly endoscopies.

This case illustrates the importance of advanced genetic testing, even in adult patients. While most monogenic intestinal diseases develop in the first years of life, several genetic disorders can present in childhood, adolescence, and even adult life. Quaranta et al. recently screened by WES 503 patients with severe IBD (defined by need for administration of biologics or surgery) above the age of 7 years and identified only one patient with X-linked inhibitor of apoptosis (XIAP) deficiency (diagnosed at the age of 41 years) (49). The patient originally presented at 11 years of age with CD, did not respond to immunomodulators and biologics and required multiple admissions and surgeries, resulting in short bowel syndrome (49). Besides the patient's suffering over the years, unnecessary procedures, and huge cost, the diagnosis provided a tailored therapeutic approach, since HSCT is curative for XIAP deficiency (50). In a different study from the same group, a deleterious G6PC3 mutation was identified in a 19-years-old patient with history of congenital neutropenia and IBD that was diagnosed in his adolescence (51). HSCT in this patient led to complete remission of both intestinal inflammation and neutropenia (51), providing another proof for the ability to provide personalized therapies based on genetic information. Similarly, several reports of LRBA and CTLA4 deficiency included patients who manifested at adulthood (42, 43), emphasizing the importance of genetic testing even in adult patients with late-onset diseases.

There were several clues that hinted that our patient had a monogenic disorder, including consanguinity, the presentation of several autoimmune disorders, the atypical enteropathy phenotype, and family history of two family members with gastric adenocarcinoma developed at a young age. Gastric adenocarcinomas have been previously reported in patients with *LRBA* and *CTLA4* mutations (41). Uhlig et al. have suggested other clinical or histologic findings that should raise the suspicion for an underlying monogenic disorder. These include young age at presentation; strong family history of IBD or immunodeficiency; atypical endoscopic or histological findings; resistance to conventional therapies; presence of recurrent infections (especially atypical ones); skin, nail, and hair abnormalities; autoimmunity; and early development of tumors (6).

The implications of reaching an accurate diagnosis in the case presented can be interpreted at different levels, including the ability to provide out-of-the-box therapies (abatacept, sirolimus, and/or HSCT), preventing additional diagnostic efforts with high costs and the ability to offer the patient genetic screening of his family and counseling. In addition, identifying more cases of this rare disease will help to improve our understanding of the clinical spectrum and the heterogeneity in age at onset of this disease.

In conclusion, the possibility of monogenic IBD-like disorder should be taken into account even in adult patients. This study highlights the importance of next-generation sequencing in cases with unique clinical phenotypes, including those beyond the young age, since it may benefit patient care and outcome.

## Data Availability Statement

The data can be found on the NCBI database—PRJNA643689. Other raw data supporting the conclusions of this article will be made available by the authors, without undue reservation, to any qualified researcher.

## Ethics Statement

The study was approved by the local IRB committee at Sheba Medical Center. Informed written consent was obtained from the patient for the studies conducted and for publication of any potentially identifiable images or data included in this article.

## Author's Note

SS and DS contributed to this research as part of the international Very Early-Onset Inflammatory Bowel Disease (VEO-IBD) Consortium.

## Author Contributions

All authors listed have made a substantial, direct and intellectual contribution to the work, and approved it for publication.

## Conflict of Interest

The authors declare that the research was conducted in the absence of any commercial or financial relationships that could be construed as a potential conflict of interest.
