# Supplementary material for: Monogenic Inflammatory Bowel Disease: It's Never Too Late to Make a Diagnosis
Source: Front Immunol. 2020 Sep 4;11:1775. doi: 10.3389/fimmu.2020.01775 (PMC7509434; doi:10.3389/fimmu.2020.01775)
Supplement: Supplementary file 4 [file Image_1.pdf]

Supplemental Figure 1

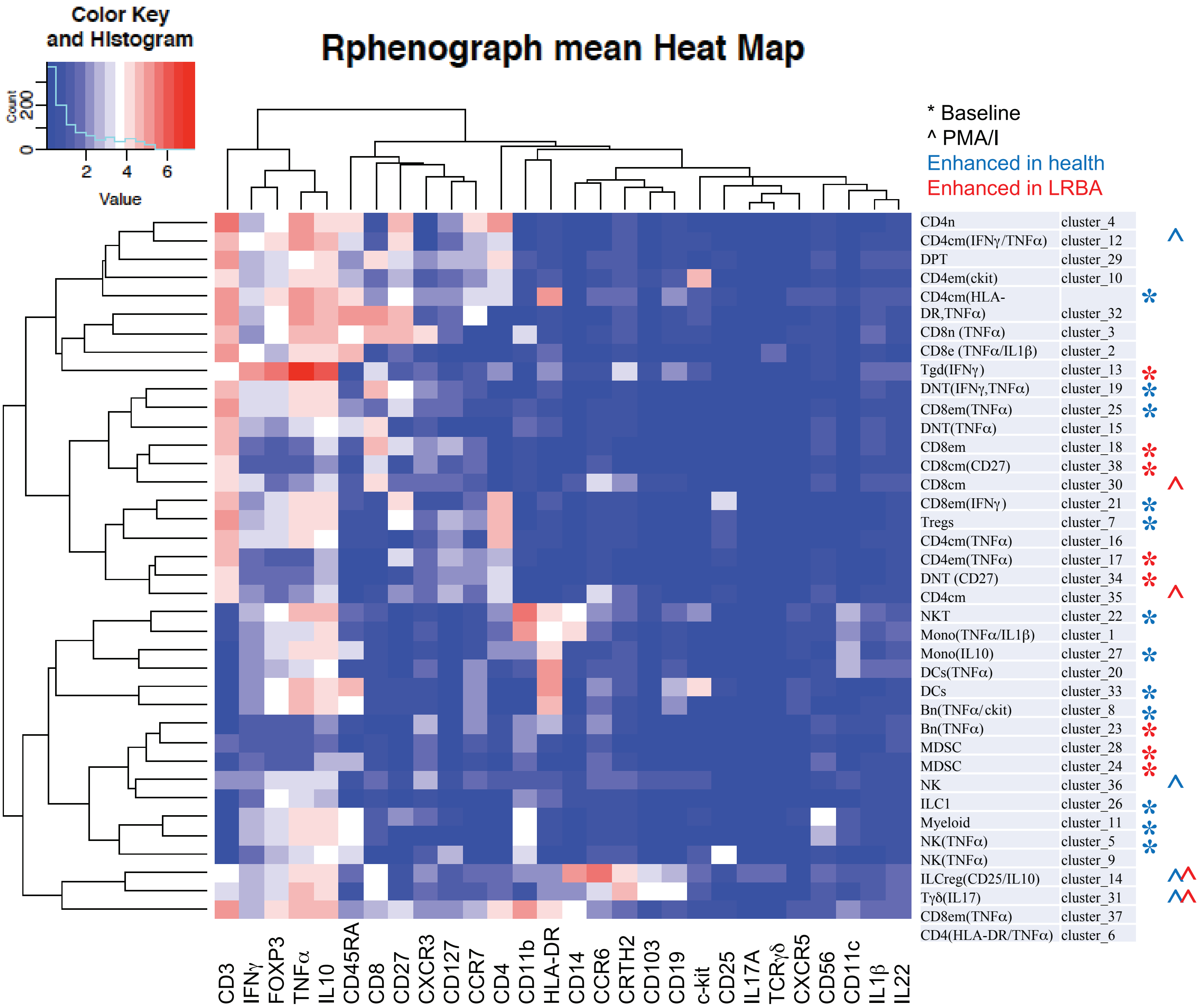

Supplemental Figure 1: Heat map demonstrating differences in immune landscape between LRBA-deficient patient and controls. Figure displays heatmap of marker expression under baseline conditions and with PMA/I stimulation in clusters associated with Figure 3B. Clusters enhanced in health are indicated with blue, those enhanced in LRBA are indicated in red. *represents populations as baseline while ^represents clusters upon PMA/I stimulation.
